# Improving Radiological Quality of Proximal Femoral Nail Antirotation Fixation for Intertrochanteric Femoral Fractures: A Closed-Loop Clinical Audit of Reduction Quality, Tip–Apex Distance, and Helical Blade Position

**DOI:** 10.7759/cureus.117309

**Published:** 2026-10-01

**Authors:** Phyo Thant, Mohamed Arsham Abdul Rasheed, Sai Tun, Phay Phone Paing, Thet Zay Yar, Nay Aung Zin

**Affiliations:** 1 Orthopaedic Surgery, University of Medicine (1) Yangon, Yangon, MMR; 2 Orthopaedic Surgery, Kulhudhuffushi Regional Hospital, Kulhudhuffushi, MDV; 3 Trauma and Orthopaedics, Royal Devon University Healthcare NHS Foundation Trust, Barnstaple, GBR; 4 Trauma and Orthopaedics, Raja Isteri Pengiran Anak Saleha Hospital​, Bandar Seri Begawan, BRN; 5 Orthopaedics and Traumatology, Kulhudhuffushi Regional Hospital, Kulhudhuffushi, MDV

**Keywords:** clinical audit, fracture reduction, helical blade position, intertrochanteric femoral fracture, proximal femoral nail antirotation, tip-apex distance

## Abstract

Background

The technical quality of proximal femoral nail antirotation (PFNA) fixation for intertrochanteric femoral fractures is influenced by fracture reduction and implant positioning. Reduction quality, tip-apex distance (TAD), and helical blade position are important immediate postoperative radiological indicators of fixation quality. This closed-loop clinical audit evaluated these parameters and assessed changes following targeted quality-improvement interventions.

Aim

To evaluate and improve the immediate postoperative radiological quality of PFNA fixation for intertrochanteric femoral fractures by comparing reduction quality, TAD, and helical blade position between baseline and re-audit cycles.

Methods

A retrospective baseline audit included 21 consecutive patients with intertrochanteric femoral fractures treated with PFNA between February 4, 2025, and February 3, 2026. Immediate postoperative anteroposterior (AP) and lateral radiographs were assessed for fracture reduction using predefined radiological criteria, TAD with correction for radiographic magnification, and helical blade position using the Cleveland index. Following review of the baseline findings, targeted quality-improvement interventions were implemented between April and May 2026. These included case-based discussion of reduction techniques, reinforcement of intraoperative TAD assessment, teaching on optimal blade positioning using the Cleveland index, and reinforcement of key PFNA technical principles. Following a post-intervention implementation period, 18 additional PFNA cases were assessed from June 2026 using the same eligibility criteria, radiological definitions, and outcome indicators. Between-cycle comparisons were performed using appropriate exact statistical tests for categorical variables and Welch's independent-samples t-test for mean TAD.

Results

At baseline, good reduction was achieved in 10/21 (47.6%) cases, TAD <25 mm in 14/21 (66.7%), and ideal blade positioning in 14/21 (66.7%), while poor reduction occurred in 4/21 (19.0%). At re-audit, good reduction increased to 12/18 (66.7%), representing an absolute increase of 19.0 percentage points, while ideal blade positioning increased to 14/18 (77.8%), an increase of 11.1 percentage points. Poor reduction decreased to 0/18 (0%). Achievement of TAD <25 mm increased modestly to 13/18 (72.2%), while mean TAD decreased from 23.6 ± 4.3 mm to 21.7 ± 3.2 mm. None of the principal between-cycle differences reached statistical significance.

Conclusion

Following targeted quality-improvement interventions, favorable changes were observed in fracture reduction and helical blade positioning, including elimination of poor reductions, while achievement of TAD <25 mm remained approximately 72%. Although the between-cycle differences were not statistically significant, the findings indicate encouraging quality-improvement trends and identify TAD optimization as an important target for subsequent audit cycles. Continued radiographic review, intraoperative attention to reduction and implant positioning, and periodic re-audit are recommended.

## Introduction

Intertrochanteric femoral fractures are among the most common fragility fractures in older adults and are associated with substantial morbidity, loss of independence, and mortality. One-year mortality following hip fracture remains considerable, particularly among older patients with multiple comorbidities [1]. With increasing life expectancy and the prevalence of osteoporosis, these injuries continue to represent an important clinical burden.

Surgical fixation is the standard treatment for most intertrochanteric fractures, with the objectives of achieving stable fixation, facilitating early mobilisation, and reducing complications associated with prolonged immobility. Both extramedullary and intramedullary fixation methods are used, although cephalomedullary devices offer biomechanical advantages, including a shorter lever arm and favorable load-sharing characteristics, particularly in unstable fracture patterns [2].

The proximal femoral nail antirotation (PFNA) incorporates a helical blade that compacts the cancellous bone during insertion, thereby improving femoral-head purchase, rotational stability, and resistance to cut-out. These characteristics may provide a particular biomechanical advantage in older patients with osteoporotic bone compared with conventional lag-screw designs [3,4]. Despite favorable outcomes reported with PFNA, mechanical complications such as cut-out and fixation failure remain important concerns. These complications are influenced by bone quality and fracture morphology as well as potentially modifiable technical factors, particularly fracture reduction and cephalic implant positioning [5].

Reduction quality is an important determinant of fixation stability. Varus alignment, residual displacement, and inadequate restoration of cortical relationships may compromise mechanical stability. Baumgaertner et al. originally described the tip-apex distance (TAD) as the sum of the distances from the tip of the cephalic implant to the apex of the femoral head on anteroposterior (AP) and lateral radiographs after correction for radiographic magnification [5]. A TAD <25 mm has traditionally been associated with a lower risk of cut-out, although subsequent studies have examined the applicability of this threshold to intramedullary devices and PFNA fixation specifically [6,7].

Helical blade position within the femoral head represents another potentially modifiable technical factor. The Cleveland index, originally described by Cleveland et al., divides the femoral head into nine zones according to the position of the cephalic implant on AP and lateral radiographs [8]. Cephalic implant position has been associated with mechanical failure, supporting careful intraoperative assessment of blade placement in addition to TAD [9].

Fracture morphology may further influence the ability to achieve satisfactory reduction and implant positioning. Unstable intertrochanteric fractures can present greater technical challenges, and previous studies have identified fracture instability, reduction quality, implant position, and other technical factors as contributors to fixation failure following cephalomedullary fixation [10-13]. These observations emphasize that successful PFNA fixation depends on the interaction between fracture characteristics, reduction quality, and accurate implant placement.

Immediate postoperative radiographs therefore provide a practical means of assessing the technical quality of PFNA fixation. At Kulhudhuffushi Regional Hospital, an initial review identified variation in reduction quality, TAD, and helical blade positioning. These findings prompted a clinical audit using predefined radiological quality indicators. Following the baseline audit, targeted quality-improvement measures were introduced, including case-based discussion of reduction techniques, reinforcement of intraoperative TAD assessment, teaching on helical blade positioning using the Cleveland index, and reinforcement of key PFNA technical principles.

This closed-loop clinical audit aimed to evaluate and improve immediate postoperative radiological quality following PFNA fixation for intertrochanteric femoral fractures by comparing reduction quality, TAD, and helical blade position between the baseline audit and re-audit cycles.

## Materials and methods

Audit design and setting

This retrospective, single-center, closed-loop clinical audit was conducted in the Department of Orthopedics at Kulhudhuffushi Regional Hospital, Maldives. The audit evaluated the immediate postoperative radiological quality of PFNA fixation for intertrochanteric femoral fractures.

The audit cycle comprised four stages: baseline assessment of predefined radiological quality indicators, review of identified deficiencies, implementation of targeted quality-improvement interventions, and re-audit using the same eligibility criteria, radiological definitions, and principal outcome indicators.

Radiological quality indicators and predefined criteria

Three principal radiological quality indicators were assessed during both audit cycles: fracture reduction quality, TAD, and helical blade position.

Reduction quality was assessed using the radiological criteria described by Baumgaertner et al. [5], based on fracture alignment and displacement. The quality-improvement objective was to increase the proportion of good reductions and minimize the occurrence of poor reductions.

TAD was measured using the method originally described by Baumgaertner et al. [5] and calculated from AP and lateral radiographs after correction for radiographic magnification. For descriptive assessment, TAD was categorized as <25 mm, 25-30 mm, and >30 mm. For the principal closed-loop comparison, TAD was dichotomized as <25 mm versus ≥25 mm, with <25 mm representing achievement of the predefined technical standard.

Helical blade position was evaluated using the Cleveland index originally described by Cleveland et al. [8]. Center-center and inferior-center positions were predefined as ideal for this audit, while all other positions were classified as non-ideal. The quality-improvement objective was to increase the proportion of cases achieving ideal blade placement.

Baseline audit and eligibility

The baseline audit included all consecutive eligible patients who underwent PFNA fixation for intertrochanteric femoral fractures between February 4, 2025, and February 3, 2026. Twenty-one patients fulfilled the eligibility criteria. Data were collected retrospectively from routinely recorded clinical information and immediate postoperative radiographs available through the hospital picture archiving and communication system (PACS).

Patients were eligible if they had an intertrochanteric femoral fracture treated with PFNA fixation and adequate immediate postoperative AP and lateral radiographs available for assessment. Patients with subtrochanteric fractures, pathological fractures, previous surgery involving the affected hip, or postoperative radiographs of insufficient quality for reliable assessment were excluded.

Fracture classification

Fractures were classified according to the 2018 AO Foundation/Orthopaedic Trauma Association (AO/OTA) Fracture and Dislocation Classification Compendium [14]. For exploratory analysis, AO/OTA A1 fractures were categorized as stable, whereas A2 and A3 fractures were categorized as unstable.

Radiological assessment

Immediate postoperative AP and lateral radiographs were evaluated using digital measurement tools available within the PACS system. All radiological assessments in both audit cycles were performed by the same single observer, an orthopedic surgeon experienced in PFNA fixation, using the same predefined criteria and PACS measurement tools. This approach was used to maintain consistency in fracture classification, reduction-quality assessment, TAD measurement, and Cleveland-zone classification across both cycles. Formal intraobserver and interobserver reliability testing was not performed. Interobserver and intraobserver reliability were not formally assessed.

Fracture reduction quality was assessed using the criteria described by Baumgaertner et al. [5]. Satisfactory alignment was defined as a normal or slight valgus neck-shaft angle on the AP radiograph and <20° angulation on the lateral radiograph. Satisfactory displacement was defined as <4 mm displacement of any fracture fragment on both AP and lateral radiographs. Reduction was classified as good when both alignment and displacement criteria were satisfied, acceptable when only one criterion was satisfied, and poor when neither criterion was satisfied.

TAD was measured according to the method originally described by Baumgaertner et al. [5] as the sum of the distances from the tip of the helical blade to the apex of the femoral head on AP and lateral radiographs. Measurements were corrected for radiographic magnification using the known implant diameter as the calibration reference.

Helical blade position was assessed using the Cleveland index [8], which divides the femoral head into nine zones according to implant location on combined AP and lateral radiographs. Center-center and inferior-center positions were predefined as ideal for this audit, while all remaining positions were classified as non-ideal.

Quality-improvement interventions

Following completion of the baseline audit, the findings were reviewed within the Orthopedic Department. Opportunities for improvement were identified in fracture reduction, achievement of TAD <25 mm, helical blade positioning, and consistency of PFNA technique. The interventions were delivered through routine departmental case-based discussions and teaching sessions between April and May 2026. As these were pragmatic quality-improvement activities, individual session duration and attendance were not prospectively standardized or recorded.

Targeted quality-improvement interventions were implemented between April and May 2026. These comprised case-based discussion of reduction techniques, reinforcement of intraoperative TAD assessment and the technical target of TAD <25 mm, teaching on helical blade positioning using the Cleveland index, and reinforcement of key PFNA technical principles.

The interventions were consultant-led and involved approximately 5-8 doctors working within the orthopedic ward and operating theatre. Practical challenges included surgical workload, emergency cases, variation in staff experience, and limited time for structured formal teaching.

Post-intervention implementation period and re-audit

Following completion of the interventions, an implementation period was allowed before reassessment to enable the reinforced technical principles to become incorporated into routine clinical practice. Re-audit commenced in June 2026.

The re-audit included 18 additional patients undergoing PFNA fixation for intertrochanteric femoral fractures. The same eligibility criteria, radiological definitions, assessment methods, and principal outcome indicators used during the baseline audit were applied to the re-audit cohort.

Statistical analysis

Data were entered into Microsoft Excel and analyzed using IBM SPSS Statistics, version 26. Continuous variables were summarised using mean and standard deviation (SD), with ranges where appropriate, while categorical variables were summarised as frequencies and percentages.

For the principal closed-loop comparison, categorical outcomes between the baseline and re-audit cycles were compared using Fisher’s exact test because of the small sample sizes and sparse cell counts. Fisher’s exact test does not produce a conventional test statistic analogous to a t-, χ²-, or F-statistic; therefore, exact two-sided p-values were reported.

Mean TAD was compared between the two independent audit cycles using Welch’s independent-samples t-test because equal variances were not assumed. The t-statistic, degrees of freedom, and two-sided p-value were reported. A two-sided p-value <0.05 was considered statistically significant.

Secondary exploratory associations between categorical radiological variables were assessed using Fisher’s exact test or the chi-square test, as appropriate. Where chi-square testing was used, the χ² statistic, degrees of freedom, p-value, and an appropriate effect-size measure were reported. Given the small sample sizes, sparse cell counts, and multiple exploratory comparisons, these analyses were considered hypothesis-generating and interpreted cautiously. No adjustment for multiple comparisons was performed.

Clinical governance and ethical considerations

This project was registered with and conducted under the Quality Improvement Department of Kulhudhuffushi Regional Hospital as a clinical audit/quality-improvement activity (Audit Reference No. KRH/QID/AUD/2026/001). Data were obtained retrospectively from routinely collected clinical records and postoperative radiographs. No alteration to routine patient care or additional intervention was undertaken for research purposes.

As the project constituted clinical audit rather than research, formal institutional ethics committee/Institutional Review Board review and individual patient consent for participation were not required under the applicable institutional governance requirements. All data used for analysis were anonymized and de-identified, and patient confidentiality was maintained throughout the audit and re-audit.

## Results

Audit cohorts

The baseline audit included 21 patients. The mean age was 71.3 ± 5.0 years (range: 63-80 years); 7/21 (33.3%) were male, and 14/21 (66.7%) were female. Ten of 21 fractures (47.6%) were classified as stable (AO/OTA A1), while 11/21 (52.4%) were unstable (AO/OTA A2/A3).

The re-audit included 18 additional patients. The mean age was 70.4 ± 5.1 years (range: 62-79 years); 6/18 (33.3%) were male, and 12/18 (66.7%) were female. Nine of 18 fractures (50.0%) were stable, and 9/18 (50.0%) were unstable. Demographic and fracture characteristics are summarised in Table 1.

**Table 1:** Demographic and fracture characteristics of the baseline and re-audit cohorts. Categorical values are presented as n/N (%) unless otherwise stated. SD: standard deviation; AO/OTA: Arbeitsgemeinschaft für Osteosynthesefragen/Orthopaedic Trauma Association.

Characteristics	Baseline (n=21)	Re-audit (n=18)
Age, mean ± SD, years	71.3 ± 5.0	70.4 ± 5.1
Age range, years	63–80	62–79
Male	7 (33.3%)	6 (33.3%)
Female	14 (66.7%)	12 (66.7%)
Stable fracture (AO/OTA A1)	10 (47.6%)	9 (50.0%)
Unstable fracture (AO/OTA A2/A3)	11 (52.4%)	9 (50.0%)

Baseline radiological quality

At baseline, good reduction was achieved in 10/21 (47.6%) cases, acceptable reduction in 7/21 (33.3%), and poor reduction in 4/21 (19.0%). Mean TAD was 23.6 ± 4.3 mm, with 14/21 (66.7%) cases achieving TAD <25 mm. Ideal helical blade positioning was achieved in 14/21 (66.7%) cases. These findings identified reduction quality, TAD optimization, and blade positioning as potential areas for quality improvement.

A representative postoperative radiographic assessment demonstrating good reduction and achievement of TAD <25 mm is shown in Figure 1. Helical blade positioning according to the Cleveland index is illustrated in Figure 2.

**Figure 1:**
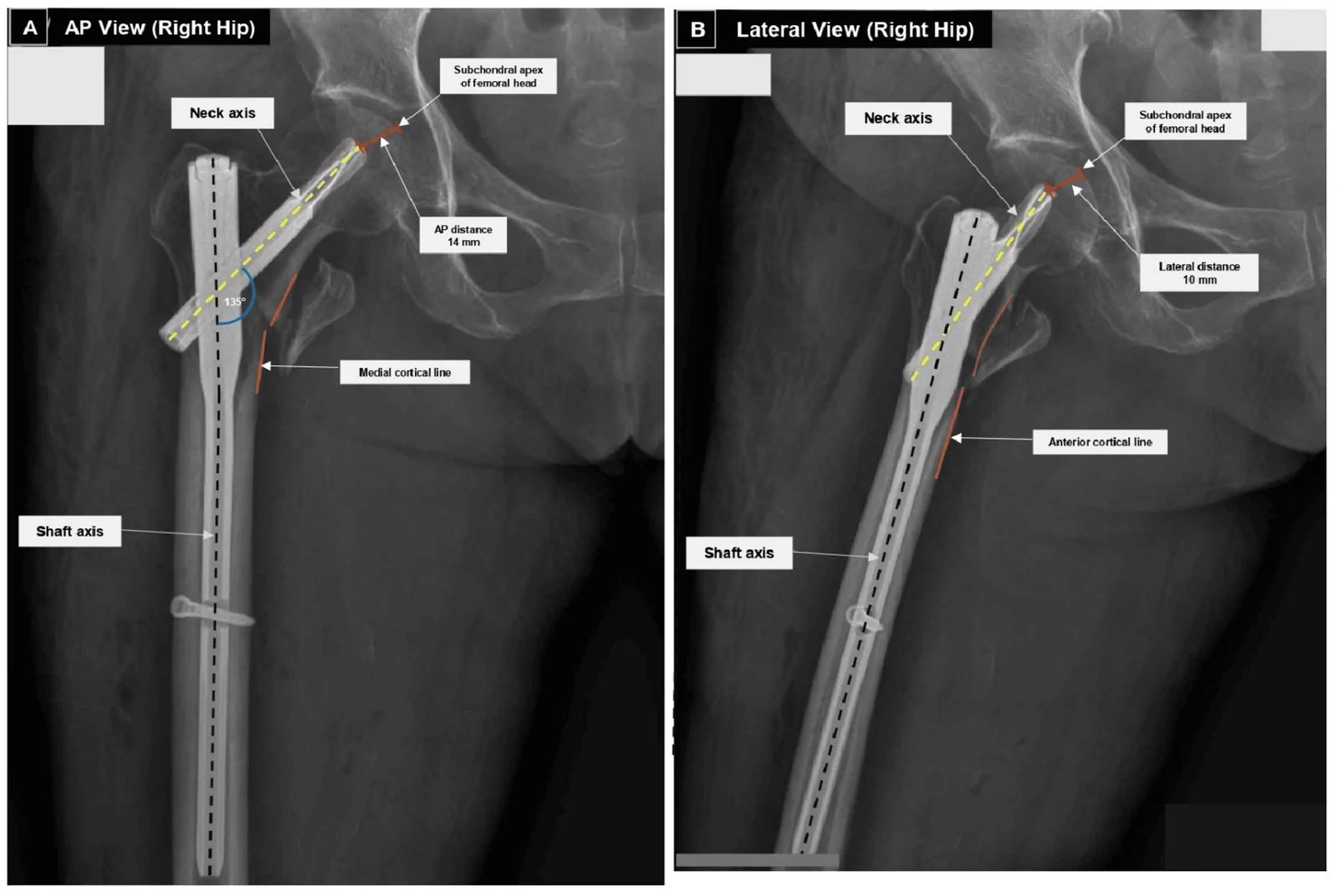
Representative postoperative radiographs demonstrating good reduction and a tip–Apex distance <25 mm following PFNA fixation. Representative postoperative radiographic assessment following proximal femoral nail antirotation (PFNA) fixation of an intertrochanteric femoral fracture. (A) Anteroposterior (AP) view demonstrates a neck-shaft angle of 135° with 2.5 mm medial cortical displacement. The AP component of the tip–apex distance (TAD), measured along the femoral neck axis from the tip of the cephalic implant to the subchondral apex of the femoral head, was 14 mm. (B) Lateral view demonstrates 2 mm anterior cortical displacement, with a lateral TAD component of 10 mm. The resulting TAD was 24 mm (14 + 10 mm). Based on the predefined audit criteria, the illustrated case satisfied the alignment and displacement criteria for good reduction and achieved the technical target of TAD <25 mm. Abbreviations: AP: Anteroposterior; PFNA: Proximal femoral nail antirotation; TAD: Tip–apex distance.

**Figure 2:**
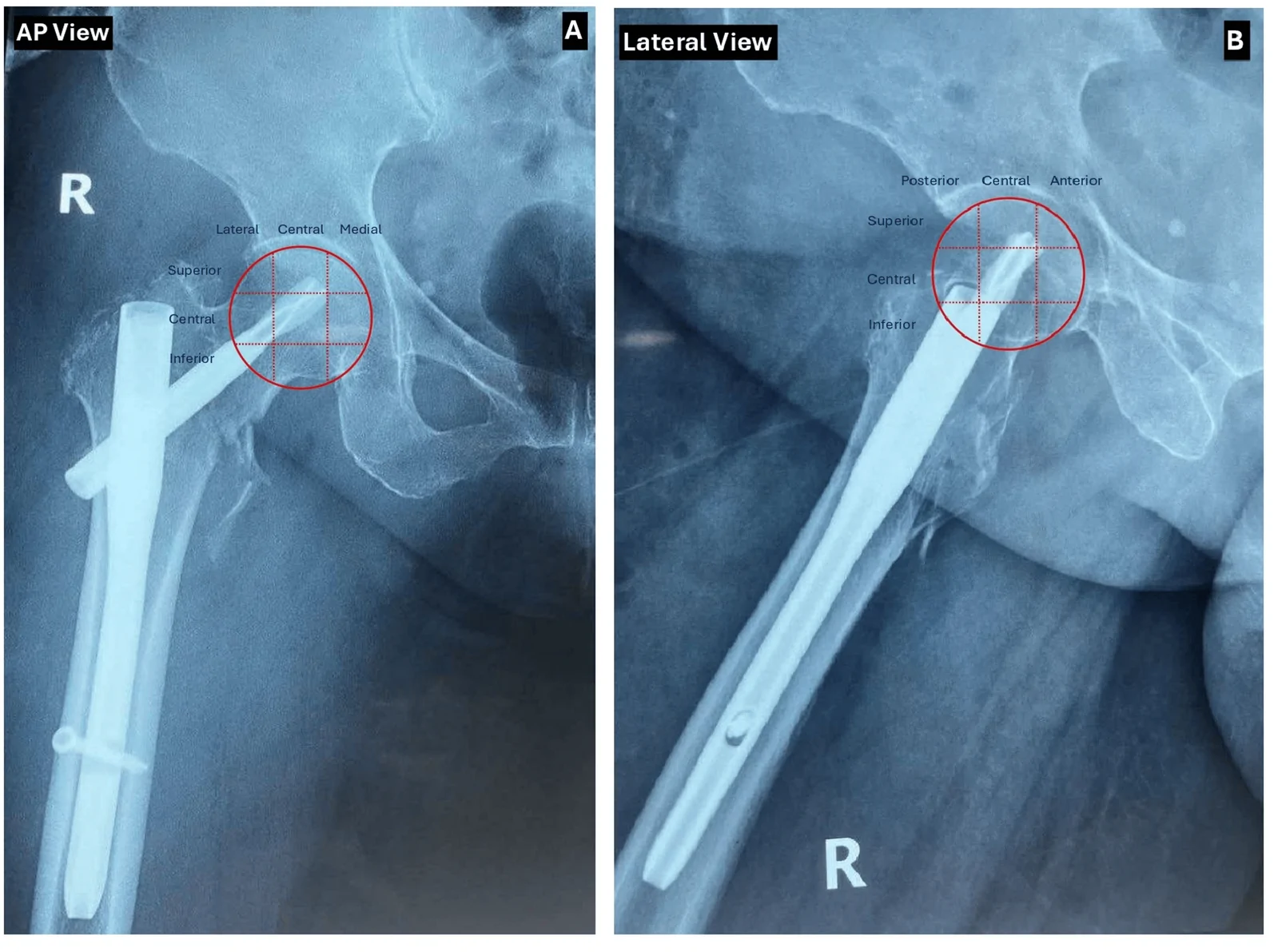
Representative assessment of helical blade position using the Cleveland Index. Representative postoperative AP (A) and lateral (B) radiographs following PFNA fixation demonstrating division of the femoral head into the nine Cleveland zones. The helical blade tip occupies the central zone on both AP and lateral views, corresponding to a center-center position. Center-center and inferior-center positions were predefined as ideal blade positions for this audit.

Closed-loop comparison

Following implementation of the targeted interventions, favorable directional changes were observed in reduction quality and helical blade positioning. Good reduction increased from 10/21 (47.6%) at baseline to 12/18 (66.7%) at re-audit, representing an absolute increase of 19.0 percentage points. Poor reduction decreased from 4/21 (19.0%) to 0/18 (0%).

Ideal blade positioning increased from 14/21 (66.7%) to 14/18 (77.8%), an absolute increase of 11.1 percentage points. Achievement of TAD <25 mm increased from 14/21 (66.7%) to 13/18 (72.2%), an absolute increase of 5.6 percentage points. Mean TAD decreased from 23.6 ± 4.3 mm to 21.7 ± 3.2 mm.

None of the principal between-cycle comparisons reached statistical significance. Good reduction (Fisher’s exact test, p = 0.334), poor reduction (p = 0.110), TAD <25 mm (p = 0.742), and ideal blade position (p = 0.497) showed no statistically significant between-cycle differences. Mean TAD was also not significantly different between cycles (Welch’s t(36.29) = 1.54, p = 0.132). The principal closed-loop comparisons (Table 2, Figure 3).

**Table 2:** Comparison of principal radiological quality indicators between baseline and re-audit cycles. Categorical values are presented as n/N (%) unless otherwise stated. Continuous data are presented as mean ± standard deviation. TAD: Tip-Apex Distance; pp: percentage points. Categorical between-cycle comparisons were performed using Fisher’s exact test because of the small sample sizes and sparse cell counts. Fisher’s exact test does not yield a conventional test statistic; therefore, exact p-values are reported. Mean TAD was compared using Welch’s independent-samples t-test, with the t-statistic and degrees of freedom reported. A two-sided p-value <0.05 was considered statistically significant. None of the between-cycle comparisons reached statistical significance.

Quality Indicator	Baseline Audit (n=21)	Re-audit (n=18)	Absolute Change	Statistical Test	p-value
Good reduction	10 (47.6%)	12 (66.7%)	+19.0 pp	Fisher’s exact test	0.334
Poor reduction	4 (19.0%)	0 (0.0%)	−19.0 pp	Fisher’s exact test	0.110
TAD <25 mm	14 (66.7%)	13 (72.2%)	+5.6 pp	Fisher’s exact test	0.742
Ideal blade position	14 (66.7%)	14 (77.8%)	+11.1 pp	Fisher’s exact test	0.497
Mean TAD, mm	23.6 ± 4.3	21.7 ± 3.2	−1.9 mm	Welch’s t(36.29) = 1.54	0.132

**Figure 3:**
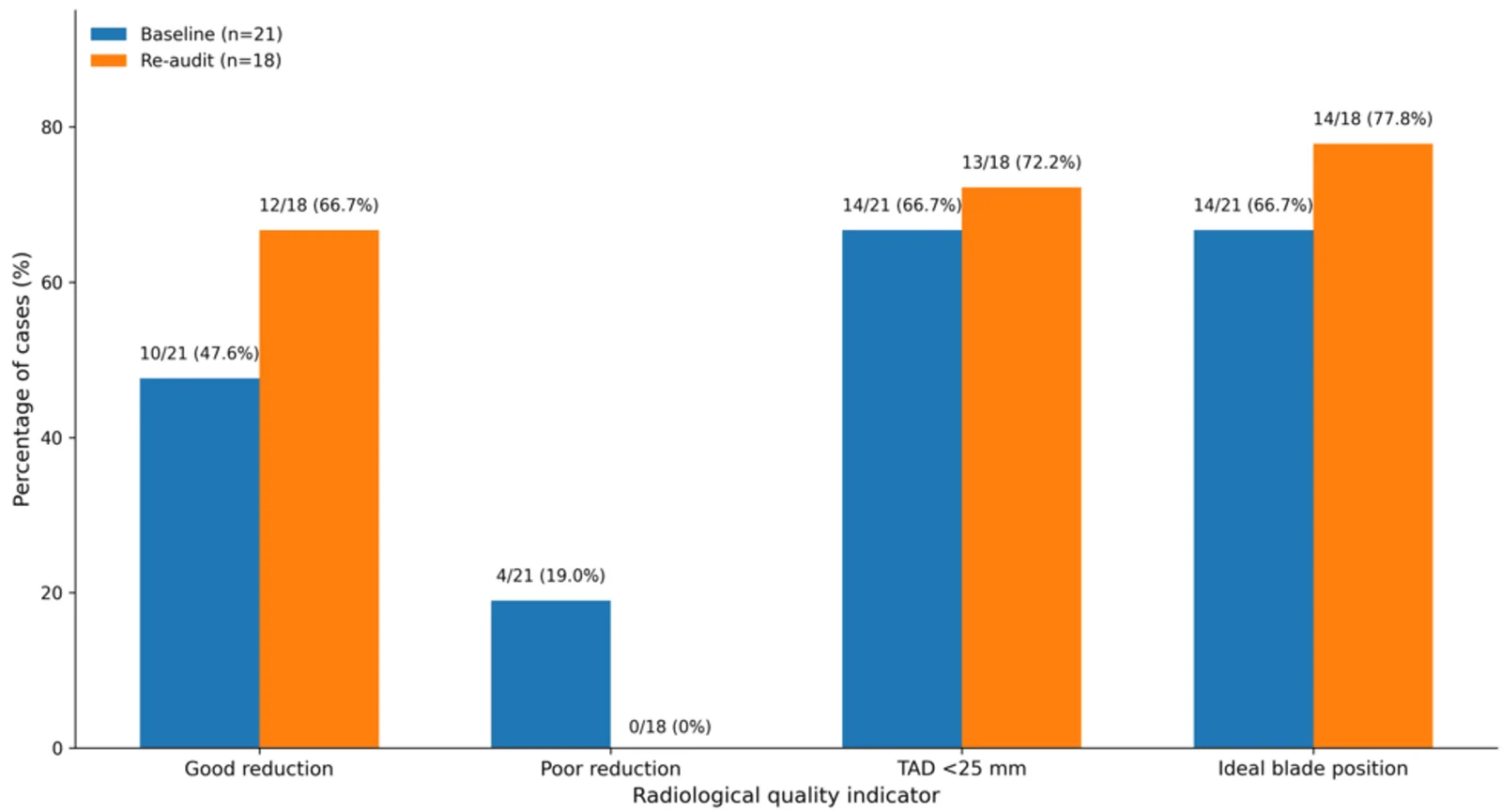
Comparison of principal radiological quality indicators between the baseline audit and re-audit. Good reduction increased from 10/21 (47.6%) at baseline to 12/18 (66.7%) at re-audit, while poor reduction decreased from 4/21 (19.0%) to 0/18 (0%). Achievement of TAD <25 mm increased from 14/21 (66.7%) to 13/18 (72.2%), whereas ideal helical blade positioning increased from 14/21 (66.7%) to 14/18 (77.8%). None of the between-cycle differences reached statistical significance. TAD: Tip-Apex Distance.

Overall, the re-audit demonstrated favorable directional changes in reduction quality and helical blade positioning, whereas TAD performance remained largely unchanged. None of the between-cycle differences reached statistical significance (Table 2, Figure 3).

Exploratory findings

Exploratory analyses suggested potential relationships among reduction quality, TAD, blade positioning, and fracture stability. Within the re-audit cohort, 9/9 (100%) stable fractures achieved good reduction compared with 3/9 (33.3%) unstable fractures. Similarly, TAD <25 mm was achieved in 9/9 (100%) stable fractures compared with 4/9 (44.4%) unstable fractures. Ideal blade positioning was achieved in 9/9 (100%) stable fractures and 5/9 (55.6%) unstable fractures.

Because of the small subgroup sizes and multiple exploratory comparisons, these findings were considered hypothesis-generating and were not used to infer an intervention effect.

## Discussion

This closed-loop clinical audit evaluated immediate postoperative radiological quality following PFNA fixation for intertrochanteric femoral fractures and assessed changes following targeted quality-improvement interventions. Good reduction increased from 10/21 (47.6%) at baseline to 12/18 (66.7%) at re-audit, ideal blade positioning increased from 14/21 (66.7%) to 14/18 (77.8%), and poor reduction decreased from 4/21 (19.0%) to 0/18 (0%). Achievement of TAD <25 mm increased modestly from 14/21 (66.7%) to 13/18 (72.2%), while mean TAD decreased from 23.6 ± 4.3 mm to 21.7 ± 3.2 mm. None of the principal between-cycle differences reached statistical significance; therefore, the findings should be interpreted as encouraging quality-improvement trends rather than evidence of a definitive intervention effect.

Adequate fracture reduction is a fundamental technical objective in intertrochanteric fracture fixation. Inadequate alignment or residual displacement may compromise construct stability and contribute to mechanical failure. Baumgaertner et al. emphasized the importance of reduction and implant positioning in reducing fixation failure [5], while subsequent studies have also identified reduction quality as an important technical factor following cephalomedullary fixation [11,12]. At baseline, good reduction was achieved in 10/21 (47.6%) cases and poor reduction occurred in 4/21 (19.0%), identifying reduction quality as an important target for improvement. Following case-based discussion of reduction techniques and reinforcement of PFNA technical principles, good reduction increased to 12/18 (66.7%), while no poor reductions were observed (0/18, 0%) during re-audit. Although these changes were not statistically significant, elimination of poor reductions represents a clinically encouraging quality-improvement finding. Given the small cohort sizes, absence of statistical significance should not be interpreted as evidence of equivalence between cycles.

TAD remains an established radiological parameter for assessing cephalic implant positioning. Baumgaertner et al. demonstrated an association between increasing TAD and cut-out and proposed <25 mm as an important technical target [5]. Subsequent studies have examined its relevance to intramedullary devices and PFNA specifically [6,7]. In this audit, achievement of TAD <25 mm increased modestly from 14/21 (66.7%) at baseline to 13/18 (72.2%) at re-audit, while mean TAD decreased from 23.6 ± 4.3 mm to 21.7 ± 3.2 mm. Although the direction of change was favorable, the difference was not statistically significant. Unlike reduction quality and blade positioning, TAD therefore did not demonstrate meaningful directional improvement following the initial intervention program. The persistence of 5/18 (27.8%) re-audit cases with TAD ≥25 mm identifies TAD optimization as a specific priority for the next audit cycle.

Helical blade position represents another potentially modifiable technical factor. The Cleveland index provides a systematic framework for describing cephalic implant location [8], and previous work has demonstrated an association between cephalic implant position and mechanical failure [9]. Ideal blade positioning increased from 14/21 (66.7%) at baseline to 14/18 (77.8%) at re-audit following teaching on the Cleveland index and reinforcement of appropriate implant placement. Although this 11.1-percentage-point increase was not statistically significant, it represents an encouraging direction of improvement.

Fracture morphology may influence the technical difficulty of PFNA fixation. Unstable intertrochanteric fractures can be more challenging to reduce and maintain during implant insertion, and previous studies have reported associations between fracture instability and adverse technical or mechanical outcomes [10-13]. In the re-audit cohort, good reduction was achieved in 9/9 (100%) stable fractures compared with 3/9 (33.3%) unstable fractures; TAD <25 mm was achieved in 9/9 (100%) stable fractures compared with 4/9 (44.4%) unstable fractures; and ideal blade positioning was achieved in 9/9 (100%) stable fractures compared with 5/9 (55.6%) unstable fractures. However, these subgroup analyses involved very small numbers and were exploratory. They should therefore be regarded as hypothesis-generating rather than evidence of an independent effect of fracture stability.

A key strength of this project was completion of the clinical audit cycle. Deficiencies identified during the baseline audit directly informed targeted interventions addressing reduction technique, intraoperative TAD assessment, Cleveland-index blade positioning, and consistency of PFNA technical principles. The same eligibility criteria, radiological definitions, and principal quality indicators were subsequently applied during re-audit. This demonstrates how routinely available postoperative radiographs can be used to identify variation in technical performance, implement targeted changes, and reassess local practice.

The observed changes cannot, however, be attributed definitively to the interventions. The baseline and re-audit cohorts were small and independent, fracture case mix differed between cycles, and other factors including fracture complexity, surgeon experience, bone quality, and operative conditions may have influenced the findings. None of the principal between-cycle comparisons reached statistical significance. Accordingly, the results represent favorable quality-improvement trends following intervention rather than proof of causality.

The findings are consistent with the broader principle that successful intertrochanteric fracture fixation depends on the interaction between fracture characteristics, reduction quality, and implant positioning. Studies comparing intramedullary and extramedullary fixation indicate that implant choice alone does not determine outcome and that appropriate surgical technique remains important [2,15]. PFNA-specific series have reported favorable outcomes, including in older patients and unstable fracture patterns [3,4,16,17]. Nevertheless, mechanical complications such as cut-out remain recognized following cephalomedullary fixation [13,18,19]. These observations support continued monitoring of modifiable technical parameters rather than assuming that implant design alone ensures satisfactory fixation.

Strengths and limitations

A principal strength of this project is its closed-loop design, with the same core radiological indicators evaluated before and after targeted quality-improvement interventions. The interventions were directly informed by deficiencies identified during the baseline audit, and predefined radiological criteria allowed consistent comparison between cycles.

Several limitations should be acknowledged. First, the sample sizes were small, comprising 21 baseline and 18 re-audit cases, which limited statistical power and increased the possibility of type II error. Multiple secondary exploratory comparisons were performed without adjustment for multiplicity and should therefore be regarded as hypothesis-generating.

Second, the retrospective design limited control over potential confounding factors, including fracture complexity, bone quality, surgeon experience, and intraoperative decision-making. The baseline and re-audit cohorts were independent rather than matched, and differences in case mix may have influenced the observed results.

Third, radiological measurements were performed by a single observer, and interobserver and intraobserver reliability were not formally assessed. Measurement variability and observer bias may therefore have influenced classification of reduction quality, TAD, and blade position.

Finally, the audit evaluated immediate postoperative radiological quality rather than longer-term clinical outcomes. Fracture union, cut-out, implant failure, reoperation, mortality, functional outcomes, and patient-reported outcome measures were not assessed. The findings should therefore be interpreted as measures of technical radiological quality rather than evidence of improved clinical outcomes. The single-center setting further limits generalisability.

Future audit cycle

The findings support continuation of the audit cycle. Future reassessment should aim to maintain the elimination of poor reductions and improvement in ideal blade positioning while specifically targeting increased achievement of TAD <25 mm. Continued case-based radiographic review, reinforcement of intraoperative TAD assessment, and focused discussion of technically challenging unstable fractures should be considered. Evaluation of a larger subsequent cohort would provide greater precision and help determine whether the observed improvements are sustained over time.

## Conclusions

This closed-loop clinical audit demonstrated favorable changes in the immediate postoperative radiological quality of PFNA fixation following targeted quality-improvement interventions. Improvements were observed in fracture reduction quality and ideal helical blade positioning, with elimination of poor reductions during the re-audit cycle. Tip-apex distance showed a more modest improvement and remains an important area for further optimization.

Although the principal between-cycle differences did not reach statistical significance, the findings demonstrate encouraging quality-improvement trends rather than evidence of a definitive intervention effect. Continued attention to fracture reduction, tip-apex distance, and helical blade positioning, together with routine postoperative radiographic review and periodic re-audit, may help sustain and further improve the technical quality of proximal femoral nail antirotation fixation.
